# Case Report: A Rare Complication Following Catheter Ablation of Scar-Related Ventricular Tachycardia

**DOI:** 10.3389/fcvm.2021.748194

**Published:** 2021-11-22

**Authors:** Xiaoyong Xu, Ming Ye, Yaxun Sun, Qiang Liu, Fusheng Ma, Chenyang Jiang

**Affiliations:** ^1^Department of Cardiology, Ningbo Medical Treatment Centre Li Huili Hospital, Ningbo, China; ^2^Department of Electrophysiology, Ningbo Medical Treatment Centre Li Huili Hospital, Ningbo, China; ^3^Department of Cardiology, Sir Run Run Shaw Hospital, School of Medicine, Zhejiang University, Hangzhou, China

**Keywords:** ventricular tachycardia, homologous ventricular separation, catheter ablation, complication, substrate modification

## Abstract

**Background:** The substrate for ventricular tachycardia (VT) in patients with structural heart disease is usually complex and often requires extensive ablation. As a result, the incidence of major procedure-related complications has been reported to be higher when compared to patients without structural heart disease. In this study, we present a rare complication after extensive substrate modification of scar-related VT.

**Case:** A 65-year-old man with ischemic cardiomyopathy was referred to the electrophysiology laboratory for radiofrequency ablation of VT following repetitive implantable cardioverter defibrillator shocks within a short period. As with hemodynamic intolerance of induced VT, an approach involving extensive endocardial substrate modification to reduce the arrhythmogenicity of the scars was adopted. After the procedure, the heart function of the patient deteriorated significantly. The postprocedural ECG showed a bizarre, extremely wide surface QRS complex (360 ms), termed as homologous ventricular separation. The pronounced dyssynchrony of the ventricle was corrected by an upgrade to cardiac resynchronization therapy with defibrillation (CRT-D). As a result, the symptoms of the patient improved significantly. The width of the intrinsic QRS complex was not recovered during an 18-month follow-up.

**Conclusion:** Homologous ventricular separation is a rare arrhythmia, manifested as two separated QRS waves. This case report demonstrates, for the first time, that homologous ventricular separation may occur after extensive substrate modification of scar-related VT. CRT-D can correct the dyssynchronous ventricle caused by homologous ventricular separation.

## Introduction

For patients with structural heart disease, catheter ablation is indicated as adjunctive treatment when recurrent ventricular tachycardia (VT) leads to repetitive implantable cardioverter defibrillator (ICD) therapies within a short time. Catheter ablation of VT has been shown to improve VT-free survival at long-term follow-up in the setting of ischemic cardiomyopathy (1). Although the complication rate of catheter ablation is higher than that of patients without structural heart disease, it is still within an accepted range (2, 3). In this study, we present a rare complication after extensive substrate modification of scar-related VT.

## Case Description

**Figure 1:**
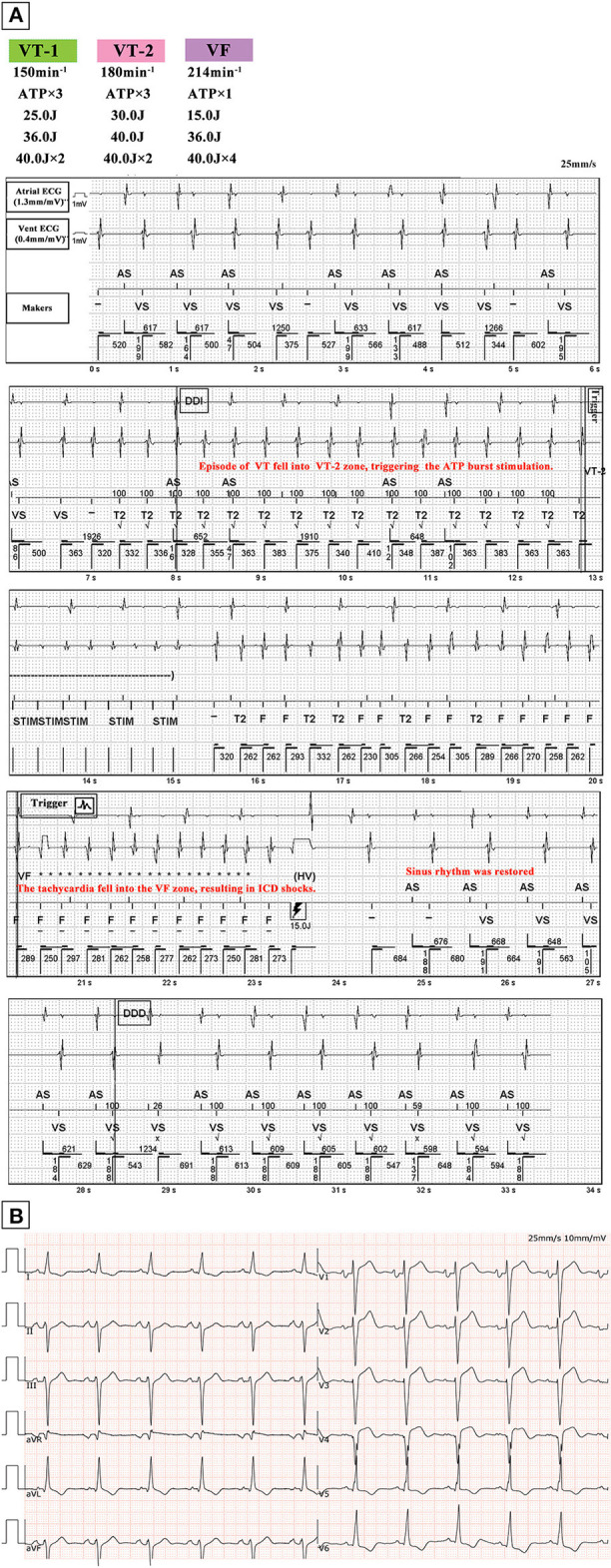
**(A)** Episode of VT detected by ICD. Sinus rhythm is restored following one attempt of ATP burst stimulation with subsequent shock application according to the ICD programming protocol shown on the upper left corner. **(B)** ECG of the patient in sinus rhythm after shock therapy, with a QRS duration of 112 ms. Abbreviations: VT, ventricular tachycardia; ICD, implantable cardioverter defibrillator; VF, ventricular fibrillation; ATP, antitachycardia pacing; vent, ventricular.

**Figure 2:**
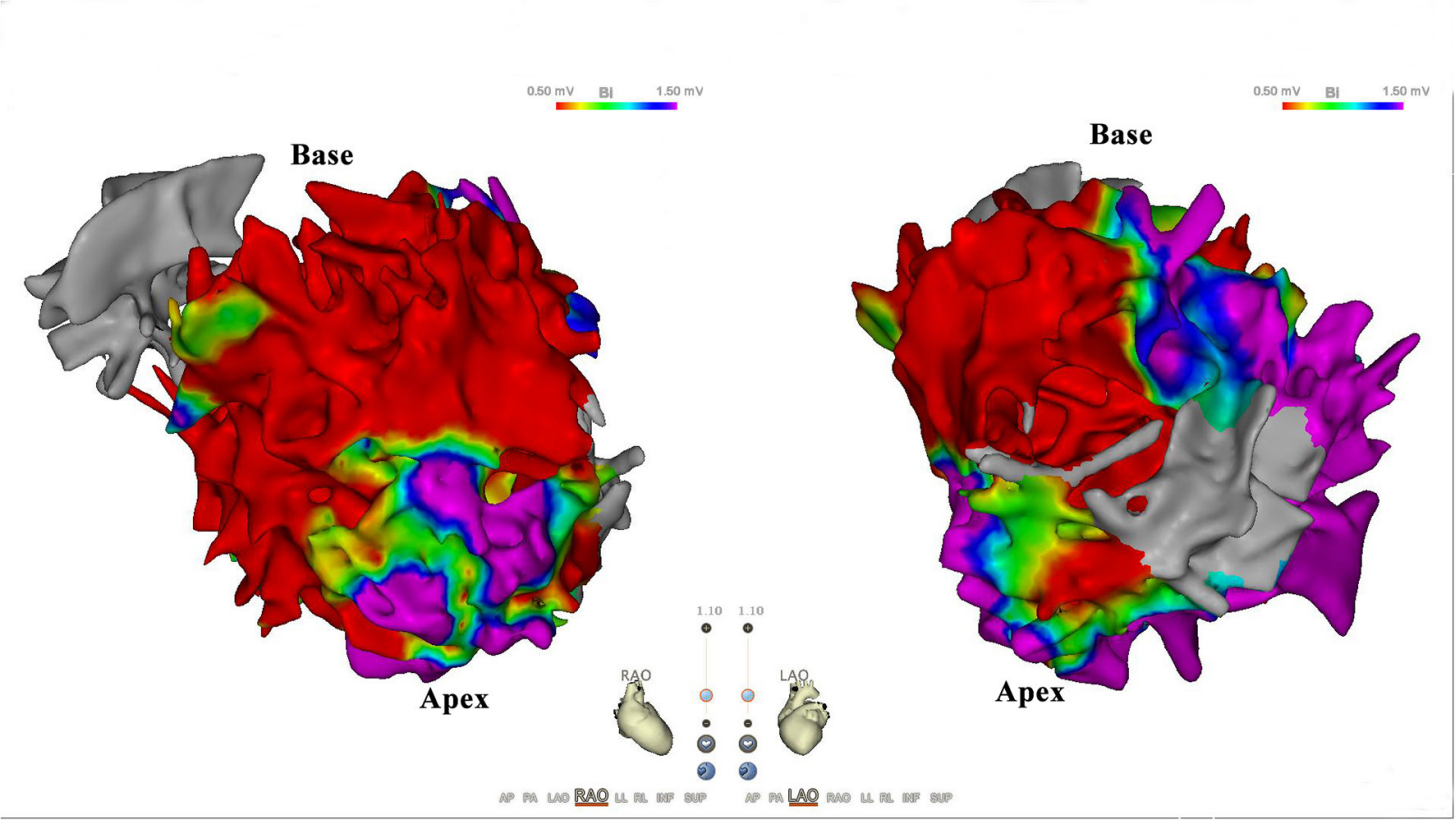
Scar distribution in endocardium. Bipolar voltage map of the left ventricular endocardium. Scar cutoff is set at <0.5 mV (red area); border zone is set at 0.5–1.5 mV. Note predilection of scar distribution to basal septal endocardium of left ventricular.

**Figure 3:**
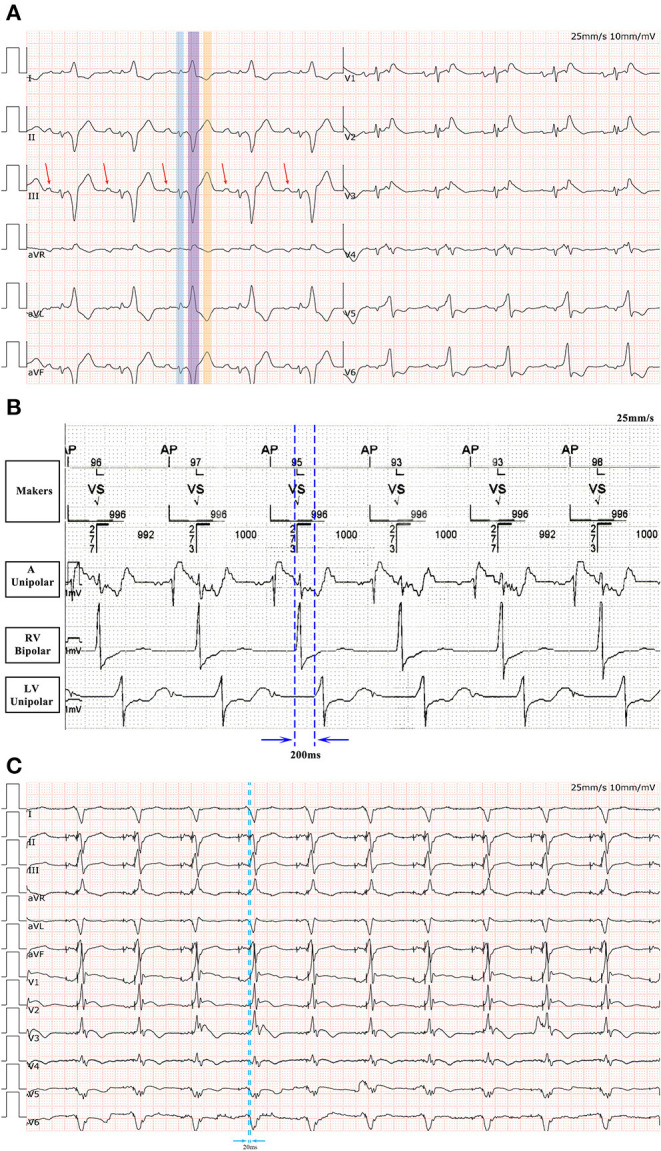
**(A)** A 12-lead ECG after ablation. The underlying rhythm is sinus rhythm with QRS duration of 360 ms. Note the initial QRS complex mimicking atrial electrical activity in standard (highlighted in blue). The real P wave is indicated by the arrow. Another QRS complex in this sequence is highlighted in purple. The wave highlighted in orange is the T wave. **(B)** The right ventricular-left ventricular interval is 200 ms in the absence of ventricular pacing. **(C)** Following ECG optimization by CRT-P programming, the QRS complex is significantly narrowed.

A 65-year-old man with ischemic cardiomyopathy was referred to the electrophysiology laboratory for radiofrequency (RF) ablation of VT following repetitive ICD shocks, even after appropriate antiarrhythmic therapy (Figure 1A). A baseline ECG showed sinus rhythm with a QRS duration of 112 ms (Figure 1B). The transthoracic echocardiogram (TTE) demonstrated a dilated left ventricle (left ventricular end-diastolic diameter of 68 mm) and left ventricular ejection fraction (LVEF) of 40%. As with hemodynamic intolerance of induced VT, an approach involving extensive endocardial substrate modification to reduce the arrhythmogenicity of scars was adopted. Endocardial electroanatomical high-density mapping was performed by using the 20-polar PentaRay catheter and the CARTO mapping system (Biosense Webster, Diamond Bar, California, USA), with the fast-anatomical mapping reconstruction resolution set at a high level. Arrhythmia substrates were identified in the basal septal endocardium of the left ventricular (Figure 2). RF current at a power setting of 40–50 W was applied to eliminate all the potential arrhythmia substrates including ventricular late potentials and local abnormal ventricular activity. The right ventricular endocardium was subsequently mapped with no low voltage area (<0.5 mV). For potential intramural arrhythmia substrate in the ventricular septum, we used bipolar RF of combining both the left and right ventricular septal surface ablation. Extensive substrate ablation was performed until: (1) no monomorphic VT was inducible and (2) only unstable VTs that were different from those previously induced. After the procedure, the heart function of the patient deteriorated significantly. The repeated TTE demonstrated an LVEF of 34%, with paradoxical motion of the left and right ventricle during both the diastole and systole. The postprocedural ECG is shown in Figure 3A. Upon close examination of the ECG, what are the unusual findings? How should the patient can be managed?

Upon close examination of the limb leads, we found a bizarre, extremely wide surface QRS complex (360 ms), which was composed of two independent QRS complexes separated by an isoelectric baseline. The first QRS complex in this sequence, highlighted in Figure 3A in a blue color, mimicked atrial depolarization in the limb leads. The second QRS complex in this sequence, highlighted in Figure 3A in a purple color, resembled a left bundle branch block (LBBB) pattern and the wave highlighted in Figure 3A in orange was a T wave.

**Figure 4:**
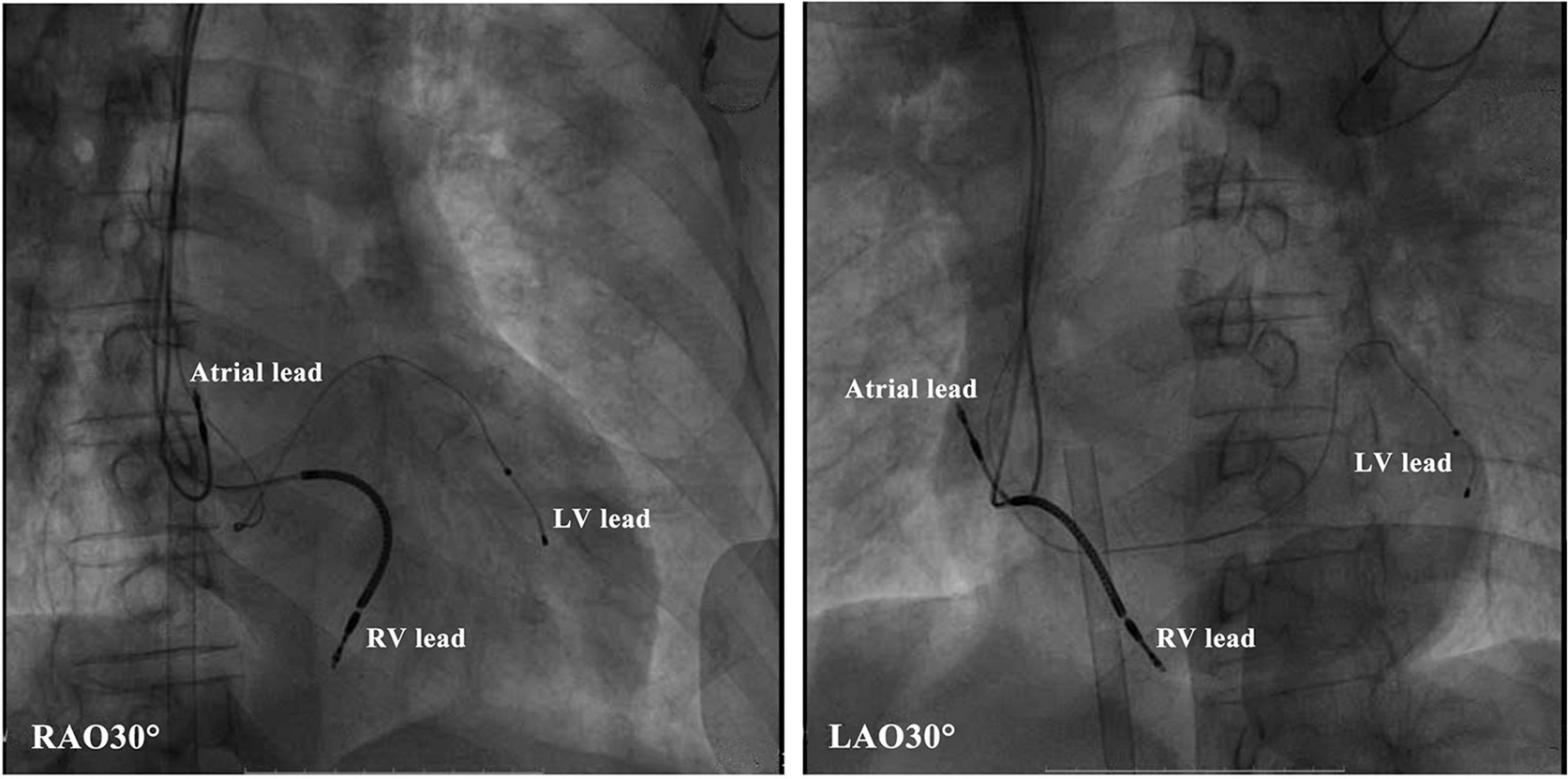
Radiologic images of the cardiac resynchronization therapy system showing lead positioning. LV, left ventricular; RV, right ventricular.

**Figure 5:**
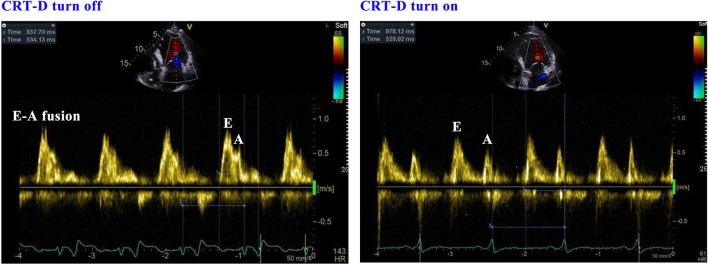
The response of echocardiography when the CRT-D is turned on and off. CRT-D, cardiac resynchronization therapy with defibrillation.

This evaluation led to the recommendation of an upgrade to cardiac resynchronization therapy with defibrillation (CRT-D). Due to the deterioration of heart function, even after appropriate heart failure therapy, the patient consented to the upgrade. The procedure was successfully performed with extracorporeal membrane oxygenation support (Figure 4). The intracardiac recordings from the right and left ventricle showed marked intraventricular conduction delay (200 ms) (Figure 3B). When the ECG was recorded simultaneously with intracardiac recording, we found that the first QRS complex in surface ECG was the result of depolarization of the right ventricle and the second QRS complex was due to depolarization of the left ventricle. Following postimplant ECG optimization, the interventricular interval was narrowed from 200 ms in intrinsic intraventricular conduction to 20 ms in biventricular pacing (Figures 3B,C). After turning on CRT-D, the fused E/A waves were separated in TTE, which suggested that the heart responded well to the CRT-D therapy (Figure 5). As a result, the symptoms of the patient improved significantly. The width of the intrinsic QRS complex was not recovered during an 18-month follow-up. The detailed timeline of the clinical course of this patient is displayed in Table 1.

**Table 1:** Detailed timeline of clinical course of the patient.

12/3/2013	The patient was diagnosed with ischemic cardiomyopathy and given the guideline-directed medical treatment of heart failure.
7/27/2017	An episode of hemodynamically unstable ventricular tachycardia (VT) resulted in treatment with implantable cardioverter-defibrillator (ICD) to prevent sudden cardiac death.
12/19/2019	The occurrence of VT electrical storm led to repetitive ICD therapies within a short period of time.
12/28/2019	The patient was referred for VT ablation. Following the procedure, the heart function of the patient deteriorated. The postprocedural ECG showed homologous ventricular separation.
1/3/2020	After an upgrade to biventricular pacing, the patient's symptoms improved significantly.
01/03/2020 to present	Follow-up period

## Discussion

Cardiac resynchronization therapy with defibrillation has demonstrated improved outcomes in selected patients with heart failure. The therapeutic benefit of CRT-D is achieved by the mechanical correction of the dyssynchronous left ventricle. Therefore, the greater the intraventricular dyssynchrony, which manifested as a widened surface QRS complex, the greater the benefit of CRT-D (4). To improve CRT-D response rates, the guideline emphasizes attention to the electrical parameters, especially before implant (i.e., LBBB and QRS duration ≥150 ms) (5). LBBB morphology is required in the class I recommendation (5). In this case, the postablation ECG shows ventricular activation separation, producing two QRS complexes that do not interfere with each other and are separated by an isoelectric baseline, resulting in pronounced ventricular dyssynchronization. However, after CRT-D corrects the dyssynchronous ventricle, the patient experienced an improvement in symptoms significantly. By means of a thorough literature review, we find that this rare arrhythmia, first reported in 1975, known as homologous ventricular separation and defined as a partial ventricular myocardium depolarized significantly later than others because of a complete myocardial conduction block (6). Homologous ventricular separation has been sporadically reported (6–9). According to previous case reports, it seems that homologous ventricular separation can occur in patients with acute deterioration of heart function (7), procainamide toxicity (6), cardiac arrest (8), and myocardial infarction (9). In the patient described in this report, ventricular separation was due to extensive ventricular ablation.

There are two strategies for the ablation of scar-related VT: (1) standard strategy: selective targeting the critical isthmus that supports the development and maintenance of VT (as identified by activation, entrainment, and pace mapping techniques) and (2) substrate modification: extensive substrate modification to reduce the arrhythmogenicity of scarring without any specific arrhythmia targeting. In a meta-analysis, Briceno et al. found that complete substrate modification results in a lower risk of long-term ventricular arrhythmia recurrence and all-cause mortality than standard ablation and incomplete substrate modification (3). In addition, the complication rate of substrate modification-related was approximately 7% including complete atrioventricular block, acute hemodynamic decompensation, puncture hematoma, cardiac tamponade, stroke/transient ischemic attack, phrenic nerve palsy, transient ST-segment elevation, and death (2, 10). To date, no homologous ventricular separation cases after substrate modification of scar-related VT have been reported. Chambers electrical isolation by catheter ablation is not uncommon. This entails isolation of the atriums from the ventricles by atrioventricular nodal ablation for rapid atrial fibrillation that cannot be controlled. In addition, the left atrium can be unintentionally isolated from the right atrium after extensive left atrium ablation (11). Due to many muscle-to-muscle connections between the right and left ventricles, it seems impossible to electrically isolate the two ventricles by catheter ablation. This case report demonstrated that ventricular separation may occur after extensive substrate modification of scar-related VT. In this case, as with hemodynamic intolerance of induced VT, we attempt to homogenize all the tissues in a low voltage area. After eliminating most of the ventricular late potentials and local abnormal ventricular activity, VT is still inducible. This suggests the presence of an intramural substrate. Empirically, we use bipolar RF of combining both the left and right ventricular septal surface ablation. During the procedure, the QRS complex suddenly separated into two parts. The potential mechanisms may include the extensive lesions in many parts of the left ventricle, especially aggressive ablation in the ventricular septum, resulting in the obstruction of electrical conduction of the ventricular muscle and Purkinje conduction system in the septum. Therefore, the spread of depolarizations from the atrium went to the right ventricle firstly, subsequently conducted to the left ventricle via apical myocardium (activation maps in sinus rhythm before and after ablation was not shown). In order to prevent this rare complication, we believe that extensive endocardial substrate modification in the ventricular septum needs to be very careful.

## Data Availability Statement

The original contributions presented in this study are included in the article. Further inquiries can be directed to the corresponding author.

## Ethics Statement

Written informed consent was obtained from the participant for the publication of this case report (including all the data and images).

## Author Contributions

XYX, MY, YXS, QL, CYJ, and FSM: contribute to conceptualization. XYX, YXS, QL, and MY: were involved in data collection, validation, and formal analysis. XYX: wrote the manuscript. All authors reviewed and approved the final manuscript for publication.

## Funding

This study was supported in part by the Natural Science Foundation of Ningbo 2017A610200 and 2014A610270 for open access publication fees.

## Conflict of Interest

The authors declare that the research was conducted in the absence of any commercial or financial relationships that could be construed as a potential conflict of interest.

## Publisher's Note

All claims expressed in this article are solely those of the authors and do not necessarily represent those of their affiliated organizations, or those of the publisher, the editors and the reviewers. Any product that may be evaluated in this article, or claim that may be made by its manufacturer, is not guaranteed or endorsed by the publisher.
